# It Takes Two to Tango: Current Understanding of the Role of M16 Family of Proteases and Their Structural Properties

**DOI:** 10.3390/biom15121697

**Published:** 2025-12-05

**Authors:** Miroslaw Jarzab, Joanna Skorko-Glonek

**Affiliations:** Department of General and Medical Biochemistry, Faculty of Biology, University of Gdansk, Wita Stwosza 59, 80-308 Gdansk, Poland; miroslaw.jarzab@ug.edu.pl

**Keywords:** M16 proteases, metalloendopeptidases, clam-shell structure, insulin degrading enzyme, mitochondrial processing peptidases, presequence peptidases, amyloid β, core proteins of *bc_1_* complex

## Abstract

The M16 protease family comprises metalloendopeptidases, characterized by a unique molecular architecture. The active enzyme molecule is composed of two halves, which together form a structure resembling a clam shell. Although the active site residues are typically located in only one half, both parts are essential for proper enzyme function. The M16 family includes many proteins that are crucial for the physiology of the organism and, therefore, are the subject of intensive research. The flagship examples are insulin-degrading enzyme (IDE), mitochondrial processing peptidases (MPPs), and mitochondrial and chloroplast presequence peptidases (PrePs). The substrates of these enzymes include many biologically important peptides, such as insulin and amyloid β. Therefore, M16 peptidases are considered attractive therapeutic targets, and understanding their structure and mechanism of action is essential for the development of specific and selective modulatory compounds.

## 1. Introduction

Metallopeptidases (metalloproteinases) are hydrolytic enzymes containing a divalent metal cation in their catalytic center, which is essential for their activity. Zinc ions are most commonly found in these enzymes, but cobalt, manganese, nickel, or copper ions may also be present. Metal ions are coordinated by amino acid ligands, usually three in number, typically His, Glu, Asp, or Lys residues [1]. According to the MEROPS protease database, metallopeptidases are divided into 16 clans [2]. Among them, the main clan is MA, comprising the largest number of metalloproteases. A characteristic feature of this clan’s active center is the HEXXH zinc-binding motif with two histidines acting as ligands to coordinate Zn^2+^ ion and the glutamate as the general base [3]. The M16 family of proteases together with the M44 family belongs to another clan, ME, which comprises metalloendopeptidases [4]. This clan is characterized by a different metal ion-binding motif, i.e., the HXXEH sequence, which is an inversion of the classic HEXXH consensus sequence found in all previously recognized zinc metallopeptidases [5].

Proteases from the M16 family were identified in various organisms from bacteria to plants and animals. The members of the M16 family play diverse biological functions, including key physiological processes. Generally, their functions can be divided into degrading and processing, which involve the breakdown or cut-off of biologically important peptides, respectively. A flagship example of the first group is the enzyme involved in glucose metabolism, insulin-degrading enzyme (insulysin, IDE). IDE was originally identified by its ability to degrade insulin, which is the preferred substrate of this enzyme. Since then, more substrates were recognized, e.g., proinsulin, glucagon, epidermal growth factor (EGF), insulin-like growth factor I and II (IGF-I, IGF-II), atrial natriuretic peptide (ANP), transforming growth factor-α (TGFα), β endorphin, amyloid β, oxidized hemoglobin, yeast propheromone, and thiolase-cleaved leader peptide [6]. Other no less important proteins are proteases responsible for cutting off (the mitochondrial processing peptidases; MPPs) or clearance of the signal peptides that direct proteins to mitochondria and chloroplasts (presequence peptidases; PreP). Mutations of the human MPP can lead to the development of severe neurological disorders with early childhood onset [7,8]. It is also worth mentioning the proteins of the *bc*_1_ complex, the core proteins. The *bc*_1_ complex is the component of the mitochondrial electron-transfer chain responsible for the electron flow from ubiquinol to cytochrome c [9]. The function of the core proteins is not clearly defined [10]. As they are not present in bacterial *bc*_1_ complexes, it has been assumed that these additional subunits have a structural rather than a functional role [11]. Finally, there is a group of M16 family members derived from protists, mainly involved in nutrient acquisition [12,13] or contributing to the pathogenicity of these organisms [14,15,16,17], and from prokaryotes, with poorly understood functions.

The physiological functions of IDE and some other members of the M16 family, as well as the prospects for using them as therapeutic targets, are the subject of a number of recent review articles [7,18,19]. Therefore, this review focuses primarily on the structure and mechanism of action of these proteases. Issues of substrate specificity and the associated challenges in developing specific modulator molecules will also be discussed.

## 2. General Structural and Functional Features of the M16 Proteases

Members of the M16 family of proteases share several characteristic properties.

**Figure 1:**
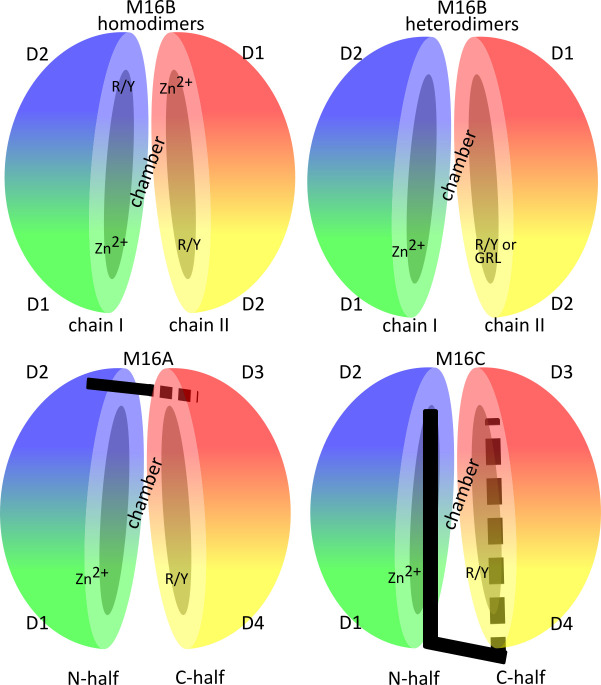
Domain organization of the M16 peptidases. Differences in the length and location of the linkers between two M16 halves are shown. Location of domains (D1–D4), the Zn^2+^-binding motif (HXXEH), the R/Y motif, and the glycine-rich loop (GRL) are indicated. Figure was created using Inkscape 1.4.2.

**Figure 2:**
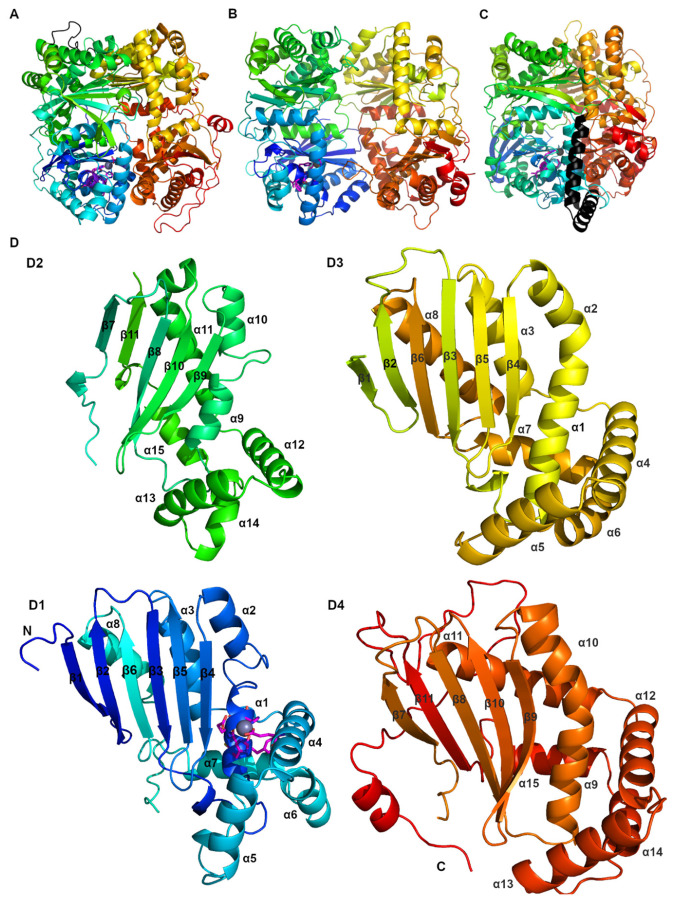
Overall structures of human IDE (**A**), yeast MPP (**B**), arabidopsis PreP (**C**), and structure homology of the four domains (**D1**–**D4**) of yeast IDE (**D**). Colors were set to spectrum rainbow (from blue to red for N- to C-terminus of the protein, respectively), linker between N- and C- halves (if present) was colored black. Figure was created using Alpha Fold 2.0 structures of IDE (AF-P14735), yeast MMP (AF-P10507 and AF-P11914 superimposed on 1HR6), and arabidopsis PreP (AF-Q9LJL3) with PyMol 1.3 and GIMP 3.0.6.

(a) The most typical feature of these peptidases is their bi-modularity, where the native molecule is formed by two parts, often referred to as “halves”. Both halves may be included in a single polypeptide chain (e.g., IDE) or they may constitute separate proteins (e.g., MPP). The presence of both halves is necessary to achieve full enzyme functionality.
(b) The overall structure of such a molecule resembles a clam shell, whose bowl-shaped halves surround a central chamber where the substrate becomes encapsulated and cleaved [20,21,22] (Figure 1).
(c) Within each half, two structurally homologous domains can be distinguished. Thus, the whole M16 molecule is composed of four domains with similar topology (Figure 1). The secondary structures of these domains in all characterized M16 proteases show a characteristic pattern: βββααββαααααβα (six β sheets and eight α helices) and ββααββαααααβ (five β sheets and seven α helices) for their N- (D1 and D3) and C-terminal domains (D2 and D4), respectively (Figure 2). Additional secondary structures can be identified within the structures of individual M16 protease members at different locations [13,22,23].
(d) The components of the active center and the regulatory sites that bind substrates are accessible from the interior of the central chamber.
(e) There are two main functional conformations: closed (associated with catalytic activity, with limited access to the central cavity) and open (allows substrate uptake or removal of degradation products).

According to their primary structures (amino acid sequences), three subfamilies within the M16 family of proteases can be distinguished, i.e., M16A, M16B, and M16C [24]. Within M16 subfamilies, other distinctive structural features were also found. These include the number of polypeptide chains building an enzyme, existence of a linker connecting these chains, and the arrangement of domains with respect to the linker [25]. Generally, proteases of the M16B family form homo- [25] or heterodimers [20,26,27,28] comprising two polypeptide chains of approximately 50 kDa, within which two domains, D1 and D2, can be distinguished, located at the N- and C-terminal ends, respectively [20,25,26,27,28]. Members of the M16A and M16C families are monomers of about 100 kDa, consisting of four domains (D1 at the N-terminus, D2–D3 in the middle, separated by linkers, and D4 at the C-terminus) [13,22,23], divided into two parts: half N (D1–2) and half C (D3–4). Those halves are connected by a linker whose length and location differentiate between M16A and M16C subfamilies (Figure 1). M16A contains a short hinge loop (~30 aa) which interacts with both domains 2 and 3 but does not contact domain 1 or 4 [22]. In contrast, the N- and C-halves of M16C are joined by a much longer (~70 aa) helical hairpin region formed by two helices that contact domain 1 and 4 and a random coil which wraps around domain 4 [13,23]. Some exceptions can also be found (e.g., FusC [29] or PqqL [30]). These peptidases are classified by MEROPS as M16B but they possess features of the M16A/C subfamilies (e.g., a linker connecting both halves of the enzyme). Structural features distinctive of M16 subfamilies are summarized in Figure 1 and Table 1.

Peptidases of the M16A and C subfamilies have similar molecular organization, exhibit similar activities, and are, therefore, considered a single functional class. They degrade a variety of peptides and polypeptides, including those having distinct secondary/tertiary structures, with chain lengths in the range of 30–70 amino acid residues (e.g., the β chain of insulin). They are referred to as “peptidasomes” by analogy with proteasomes [21]. Eukaryotic peptidases of the M16B class represent a separate functional class. These enzymes cut short N-terminal sequences that direct proteins to mitochondria or chloroplasts. For this reason, they are referred to as “processing peptidases”. Prokaryotic M16B subfamily members are more difficult to functionally categorize; they might be the most diverse in types of recognized substrates, as in most cases, only nonphysiological substrates of varying lengths were tested. Nonetheless, it was suggested that prokaryotic M16B proteases fit into the “peptidasomes” category [25].

## 3. Activity-Related Structural Motifs

In general, the M16 family has been reasonably well characterized structurally. The crystal structures of several representatives of each subfamily have been solved (summarized in Table 1). IDE is the most thoroughly characterized M16 protease in this regard. Crystal structures of IDE are available both without ligand and with bound substrates. In addition, structural studies were also conducted using other techniques, such as cryo-electron microscopy (cryo-EM) and small-angle X-ray scattering (SAXS) [43,45,47]. Analysis of these structural data allowed to distinguish and characterize the most important features of the catalytically important motifs. Some of these motifs are located in the other half than the one containing the active site. This fact largely explains the requirement for the presence of both parts to obtain the proper functionality of the M16 protease.

### 3.1. Metal-Binding Motif

**Figure 3:**
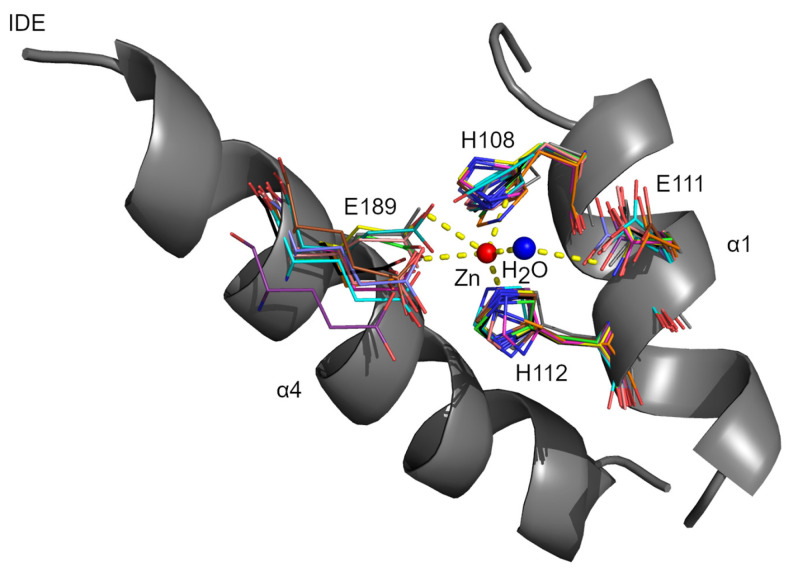
Superposition of catalytically important residues of rat IDE (gray, 180HFCEH112-E189, PDB code 3P7L) with conserved corresponding residues of yeast MPP (salmon, 1HR6), *Escherichia coli* pitrilysin (slate, 1Q2L), *Halalkalibacterium halodurans* BH2405 (green, 3HDI), bovine cytochrome *bc_1_* complex core I protein (cyan, 2A06), *Plasmodium falciparum* FLN (lightmagenta, 4NGE), human PreP (yellow, 4L3T), *Bacillus thermotolerans* AlbF (black, 7Y8U), *Sphingomonas* sp. SPH2681 (teal, 3AMJ), *Escherichia coli* PqqF (brown, 5CIO), *Pectobacterium atrosepticum* FusC (deepsalmon, 6B05), *Serratia* sp. PqqL (violetpurple, 6OFS), and *Thermus thermophilus* TTHA1264 (orange, 3EOQ). The water molecule involved in the coordination of the zinc ion (red) is shown as a blue sphere. Figure was created using PyMol 1.3 and GIMP 3.0.6.

The HXXEH motif is the core of the active center. The His residues of the motif and the separate distal Glu residue, placed in the α4 helix of D1, coordinate the zinc ion. The Glu residue within HXXEH acts as a base catalyst on the water molecule that occupies a fourth coordination site of the zinc ion (Figure 3) [20,31,32,90]. Despite inversion of the HEXXH motif sequence, the mechanism of a substrate cleavage is probably similar to that of thermolysin, the type-example of clan MA [20]. In the case of IDE, the zinc-binding site is created by a ^108^HFCEH^112^ motif and E189. The key element is the water molecule coordinated to the zinc ion and polarized by E111, thereby allowing it to carry out a nucleophilic attack on the carbonyl carbon of the peptide bond of the substrate [20]. The superimposed structures of M16 proteases show a very similar architecture of the active site with a very similar arrangement of residues involved in zinc ion coordination and water molecule activation (Figure 3) [32]. Most of the M16 members contain one HXXEH motif. It is located within the N-terminal domain (D1) of one of the polypeptide chains of M16B heterodimers or in the N-terminal domain (D1) for members of the M16A and M16C families. The exception is M16B homodimer, where both halves (subunits) are identical, so the molecule contains two HXXEH motifs [25] (Figure 1). There are also examples of M16 family members devoid of HXXEH motif, which renders them proteolytically inactive (e.g., yest core I protein) [62], or with a modified motif (e.g., bovine core I (YFVEH), which has a low activity after additional detergent treatment) [48].

### 3.2. The R/Y Pair Motif

**Figure 4:**
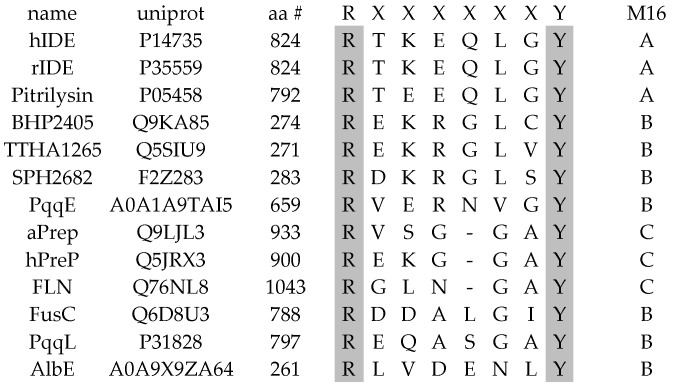
Alignment of the RY motifs of the exemplary M16 family members. Alignment was run using Clustalx2 2.1. Numbers to the left side of the sequence (aa #) indicate positions in proteins, letters (A, B, C) in the right column indicate the M16 subfamily. Identical residues among the members are shaded.

The arginine/tyrosine (R/Y) pair is located in D4 within the α10-β9 loop. This motif plays an important role in substrate binding, completing the active site and stabilizing the transition state during the hydrolysis of a peptide bond [21,22]. The R/Y pair is conserved among the M16 enzymes (Figure 4), and it occurs in almost all M16 family members (M16A, M16C, and most members of the M16B subfamily), except MPP and the core proteins of *bc*_1_ complex.

### 3.3. Glycine-Rich Loop

**Figure 5:**
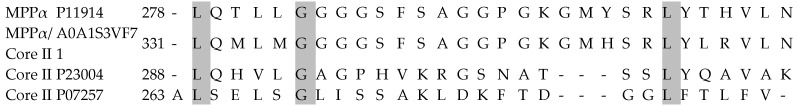
Alignment of the glycine-rich loop sequences of the MPP and core II proteins from various sources. Alignment was run using Clustalx2 2.1. Numbers to the left side of the sequence indicate positions in proteins. Identical residues among the members are shaded.

The MPP (α-subunit) [7,25] and core II proteins of *bc*_1_ complex [20], that lack the R/Y pair, have a different element, termed the glycine-rich loop (GRL). This motif is located in D4 between α9-α10 helices and is highly conserved among eukaryotic M16B peptidases (Figure 5). The glycine-rich loop is important for substrate binding [20,91,92,93] and probably also for product release (due to its flexibility) [20]. It was also suggested that the substrate remains in contact with the GRL and that its conformational flexibility is crucial for the substrate movement towards the MPP active site [92]. As the GRL is observed in close proximity to the active site, it may limit the access of substrates to the active site [20]. At the same time, it allows the MPP to adopt a partially open conformation [25], acting as a precisely balanced structural element regardless of the presence or absence of a substrate in the active site [92].

## 4. Substrate Specificity

Many M16 peptidases cleave a variety of substrates that differ significantly in amino acid sequence, length, and/or structure. Furthermore, individual enzymes digest more than one substrate, often many different substrates. For example, tomato plinsulysin cleaves insulin, systemin, substance P, bradykinin, melittin, and glucagon [94]. In yeast, peptidases Axl1 and Ste23 are involved in the production of the a-factor mating pheromone; Ste23 was also shown to cleave amyloid β and insulin [95] and is required for efficient degradation of presequence peptides resulting from the activity of the processing protease MPP in the mitochondrial matrix [96]. Mouse nardilysin cleaves glucagon [97] and polo-like kinase 3 (Plk3) to generate an activated form of Plk3 that is important for the suppression of pancreatic ductal adenocarcinoma [98] and contributes to the generation of cytotoxic T lymphocytes epitopes [99]. How are these substrates recognized? This issue has been well-studied in models of the degradative peptidase IDE and the processing peptidase MPP.

### 4.1. Substrate Recognition by IDE and Other Examples of Peptidasomes

Structural and biochemical analyses of IDE identified several structural features of the catalytic chamber which determine the specificity of the enzyme. These include IDE’s chamber size, shape, and charge complementarity [22,37,38,100]. The size of the IDE catalytic compartment (~16,000 Å^3^ [100,101]) determines the size of the substrate, which is limited to peptides less than 80 amino acids in length [100,101,102]. These properties are consistent with the size of the main known IDE substrates (listed in Table 1). Larger peptides, such as transforming growth factor-β and proinsulin, are degraded considerably slower than smaller hormones, like TGF-α and insulin [22]. The reported sizes of cavities of the other M16 proteases vary from 9400 Å^3^ for *Serratia* sp. strain FS14 PqqF, which can accommodate a relatively small substrate, like PqqA (25-residue polypeptide) [31], 10,000 Å^3^ for *Bacillus halodurans* BHP [25] or arabidopsis Prep [21], 12,500 Å^3^ for *Plasmodium falciparum* FLN, 13,300 Å^3^ for human PreP with the upper limit for substrate size determined to be 65 residues [23], 18,000 Å^3^ for *Bacillus thermotolerans* AlbF/AlbE [27], up to 21,000 Å^3^ determined for *Sphingomonas* sp. strain A1 SPH2681/SPH2682 [26].

The charge distribution within the catalytic compartment is not uniform in IDE. The inner chamber surface is formed by all four domains: in IDE-N, it is mostly neutral or negatively charged, while the surface provided by IDE-C is mostly positively charged [22,32]. Substrates that make substantial contacts with positively charged IDE C-half, like insulin β chain, amyloid-β, amylin, atrial natriuretic peptide, glucagon, and IGF-II, are excellent IDE substrates; however, brain natriuretic peptide, glucagon-like peptide, and insulin growth factor I, that have many positively charged residues, are poor IDE substrates [22]. Good charge complementation of the IDE catalytic chamber was also observed with ubiquitin as a substrate [36]. In addition to charge complementarity, another factor affecting substrate binding may be the dipole moment of the substrate molecule. For example, IGF-II, which has a pronounced dipole charge distribution that can complement the charged IDE catalytic compartment, is a better substrate compared to IGF-I, whose surface charge distribution does not harmonize well with the IDE compartment [37]. The negative electrostatic potential and shapes of other M16 proteases near to the active sites are similar, but beyond the immediate environment of the active site, the charge distribution and architecture of the peptidasome chambers are highly variable [25].

**Figure 6:**
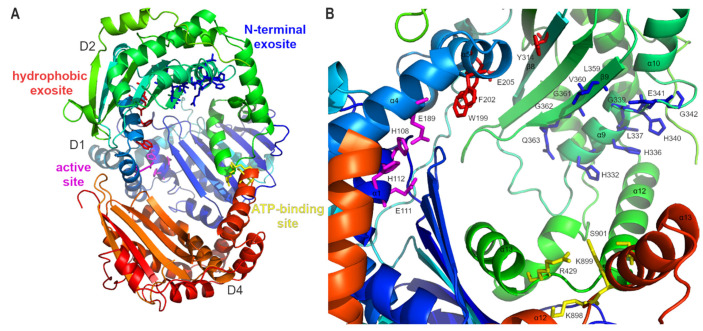
IDE exosites in relation to active site. (**A**) Overview of main known activity regulating structural elements of IDE (3CWW). Location of three subdomains (D1, D2 and D4) is indicated, and subdomain D3 was omitted for clarity. (**B**) Amino acid residues within IDE structure, important for specific IDE function or interaction with substrates. Figure was created using PyMol 1.3 and GIMP 3.0.6.

In addition to the catalytic site, IDE contains additional sites involved in substrate binding, termed exosites [22,37,45,103] (Figure 6). Noteworthy is the N-terminal exosite located in domain 2 (D2) at a distance of about 30 Å from the zinc ion of the catalytic center (HXXEH motif). This exosite was defined in the crystal structure of IDE in complex with different substrates. The proposed role for this exosite is to anchor the N-terminus of the substrate to ensure its proper positioning in the catalytic chamber [22,32]. This kind of arrangement was observed for insulin [22,46,47,100], amyloid β (1–40), amylin [22,37], IGF-II, TGF-α [37], ubiquitin [36], CCL4 [39], and glucagon [22,44].

An important phenomenon occurring in the IDE crypt is the modulation of the substrate structure. In particular, there is an induction of the formation of short β-strands in the substrate and their binding by the enzyme to form antiparallel β-sheets [22,45]. As a result, the substrate binds strongly at two sites of the enzyme: the N-terminal end of the substrate binds to the β9 chain (Figure 2D) within a specific N-terminal exosite (Figure 6B) [22,103], and the induced C-terminal β strand interacts with the β4 chain within the hydrophobic exosite near the active center, enabling substrate recognition [37,45,103]. Binding to the extended β-strand conformation of substrates seems to be universal to most types of proteases. This action (1) protects folded proteins from unintentional degradation, (2) reduces the energy required to break peptide bonds, (3) promotes better intermolecular bonding with an enzyme, and (4) increases accessibility of cleavable (unfolded) regions of substrates [104]. In the M16 protease family, the phenomenon of the short β-strand formation within the substrate and the subsequent formation of extensive, antiparallel β-sheets with the enzyme has many consequences. It is believed that it provides a sequence-independent recognition mechanism and positions the scissile bond within the catalytic site [13]. Furthermore, β-sheet pairing may stabilize the closed (active) conformation of the protease, enabling proteolysis [102].

### 4.2. Substrate Recognition by the Mitochondrial Processing Peptidase (MPP)

The MPP substrates comprise various proteins that are directed to the mitochondria by signal peptides. The N-termini of these proteins contain basic amino acids and lack negatively charged residues, which gives them a net charge of +3 to +6; this makes them ideally suited for the negatively charged MPP chamber [105]. The signaling sequences are poorly conserved at the amino acid level and vary significantly in length (15–60 aa). Nevertheless, they have some common features: a conserved arginine residue at the P_2_ position, which fits into a negatively charged substrate pocket in the MPP active site [106], and amino acids at the N-terminus that follow the bacterial N-terminal rule to stabilize the protein [107]. In addition, mitochondrial signaling sequences have the ability to form amphiphilic α-helices [20,107]. These structures are important for interacting with mitochondrial import components and, at the same time, are flexible enough to fit into the active site of the MPP [20]. In most cases, the MPP cleaves substrates at a single specific site [20], but some mitochondrial preproteins undergo a two-step processing by the MPP [106].

### 4.3. Substrate Recognition by Presequence Peptidases (PreP)

Presequence peptidases (PrePs) are present in mitochondria and chloroplasts. Their task is to remove presequence peptides which are generated by the MPP or other peptidases [108,109]. Free presequence peptides may cause many adverse effects in the cell. They can penetrate mitochondrial membranes, leading to a loss of the membrane potential, interact with membrane lipids and cause membrane rupture in chloroplasts, or inhibit preprotein maturation by inhibition of the processing enzymes in both organelles [106]. Therefore, the mitochondrial signal peptides must be degraded to non-toxic or exportable fragments by PreP oligopeptidase [110]. Human PreP (hPreP) was also shown to efficiently degrade amyloid-β [111] which makes it another M16 family member implicated in the development of Alzheimer disease [90].

**Figure 7:**
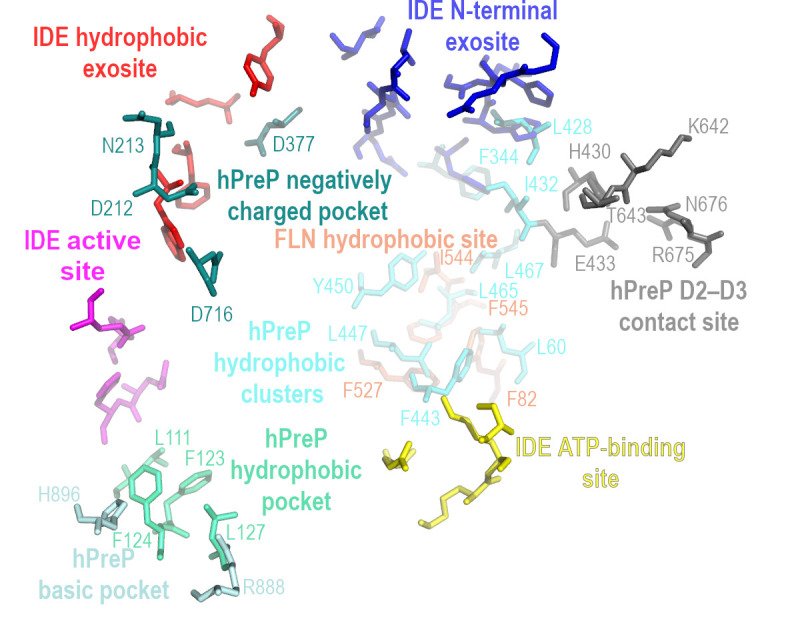
Location of catalytically important hPreP sites: negatively charged pocket (deepteal), basic pocket (palecyan), hydropohobic clasters (cyan), hydrophobic pocket (green-cyan), D2–D3 contact site (gray), and FLN hydrophobobic sites (orange) in relation to active site residues of IDE and pockets identified in IDE. Figure was created using PyMol 1.3 and GIMP 3.0.6.

Crystal structures of hPreP in complex with amyloid β allowed to identify several pockets within the N-half of its chamber. Of particular note is the hydrophobic pocket immediately adjacent to the active site (with residues L111, F123, F124, and L127) which forms an S_1_ or S’_1_ and the basic pocket (residues R888 and H896) that binds substrate C termini via salt bridges. Additionally, a hydrophobic cluster formed by residues L60, F344, L348, Y380, Y383, L428, I432, F443, M446, L447, and Y450 (Figure 7) facilitates the recognition of substrates [23]. Its residues were shown to bind amyloid β, favoring the closed state of the enzyme and supporting the transition between the closed and open forms during the proteolytic cycle of hPreP [89]. Human PreP specificity is also explained by specific electrostatic properties of certain regions of the protein, such as a negatively charged surface (D212, E213, D377 and D716) placed 15–19 Å away from the active site, which attracts positively charged residues in presequences, or hydrophobic clusters present inside the hPreP-N chamber, which facilitate the recognition of hydrophobic patches within substrates (Figure 7) [23].

## 5. Mechanism of Catalysis and Regulation of the Exemplary M16 Proteases

All M16 family members have a similar domain organization, and analysis of the available crystal structures of these proteases reveals two basic structural states, i.e., closed (aPrep [21], BHP [25], FusC [29], all with bound peptides), or open (pitrilysin (PDB: 1Q2L), PqqL [30]) conformations. This fact indicates the necessity of transitions between these states during the catalytic cycle of the enzymes. Indeed, SAXS analysis demonstrated that in solution, M16 family proteases exist in a dynamic equilibrium between closed and open states, e.g., BHP [25], IDE [43,45], hPreP [23], FusC [29], PqqL [30]. Additionally, distinct intermediate states (such as partially open or closed) were observed when analyzing cryo-EM structures [47,89]. In the case of processing proteases (eukaryotic M16B subfamily), e.g., the MPP [20] or core proteins building the cytochrome *bc*_1_ complex, a partly open conformation has been detected. This state results from the presence of the glycine-rich loop which prevents complete closure [11], regardless of the presence or absence of a substrate [11,20]. It is also speculated that GRL is involved in recruiting the substrate presequences in the active site or the release of the product [20].

The mechanisms of substrate recognition, binding, and degradation have been studied most thoroughly using the IDE protein model, and these mechanisms will be presented in detail. Analysis of available crystal structures of free IDE [32,33,41,45], with bound substrates [22,32,34,35,36,37,39,44,46,100] or with small molecules [34,40,41,42,43,44,112], revealed its closed state (IDEc). IDEc cannot load a new substrate, and the cleaved products cannot exit, so the chamber needs to undergo a significant open–closed transition during its catalytic cycle [22]. The open-state IDE (IDEo) structure was not available until 2018 when cryo-EM structures of IDE bound with Fab_H11-E_ (IDE-specific fragment of the antigen-binding region with a rigid elbow region between the heavy and light chains of Fab_H11_) allowed the observation of three apo-IDE dimer structures derived from combinations of the IDE partial open (pO) and open (O) states (O/pO dimer is dominant). Insulin-bound IDE adopting a partially closed (pC) state was also observed [47]. The existence of IDEo in solution and dynamic equilibrium between closed and open states was determined using SAXS analysis [43,45,47]. SAXS data also showed that small compounds [43] and substrates [47] promote IDE’s shift from the open to the closed state in solution. There are data indicating key structural elements and factors that influence the equilibrium between closed and open states of IDE. One specific part of the M16 crypt responsible for closed state signaling may be the exosite, which promotes the open to closed transition upon interaction of IDE with small molecules [42,43]. The closed state is also stabilized by the electrostatic attraction between the two halves of the enzyme. Therefore, the shift to the open state can be achieved by binding of anionic molecules [34]. This result was further confirmed by several structural analyses of recombinant IDE in the presence or absence of ATP [32].

The mechanism of insulin degradation has been most thoroughly studied [47,100]. Insulin and other substrates undergo conformational changes inside the catalytic chamber of IDE [22,47,100]. IDE cleaves its substrates at one [35,113], two [36], or most commonly, at multiple sites [22,37,100]. IDE initiates insulin degradation by a single cleavage of the A-chain and at least one cut in the middle of the B chain, forming fragments that contain either N-terminal or C-terminal portions of insulin. The resulting fragments are cleaved further at the known cleavage sites of insulin B chain [100]. After cleavage, the N- and C-terminal fragments of insulin permit the opening of IDE, which allows the release of products [100].

**Figure 8:**
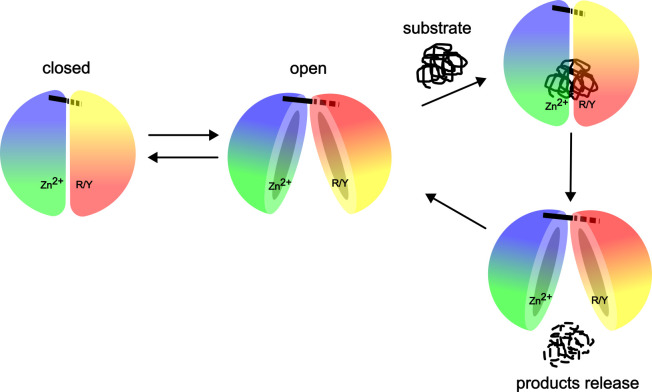
The general model for substrate binding, cleavage, and release of products proposed for peptidasomes. In solution, the M16 family proteases exist in a dynamic equilibrium between closed and open states. The substrate binds to the enzyme in its open state and induces enzyme closure which in turn allows for catalysis. Once substrate is degraded, opening of the enzyme is possible and cleavage products can be released. In the presence of the substrate, the catalytic cycle is repeated. Alternatively, the enzyme reaches equilibrium between the open and closed states when substrate is not available. Figure was created using Inkscape 1.4.2.

Based on early and recent observations, a general model for substrate binding, cleavage, and release has been proposed for peptidasomes (M16A/C and prokaryotic M16B subfamilies) (Figure 8). In solution, the M16 family proteases exist in a dynamic equilibrium between closed and open states [23,25,29,30,43,45]. In the first step, the substrate binds to the enzyme in its open state [25]. Once the substrate is bound, it induces a switch from the open conformation to the closed state, so that catalysis can occur [21,22]. This transition may be dependent on specific interaction of exosites with the hydrophobic residues of the substrate. After cleavage, the product peptides induce the opening of the enzyme, the products and substrate exchange, and the next catalytic cycle can be initiated [100] (as in the case of human PreP) [89]. Similar conformational changes can be induced by small molecules, as reported for IDE [43].

### 5.1. IDE’s Swinging Door Mechanism

**Figure 9:**
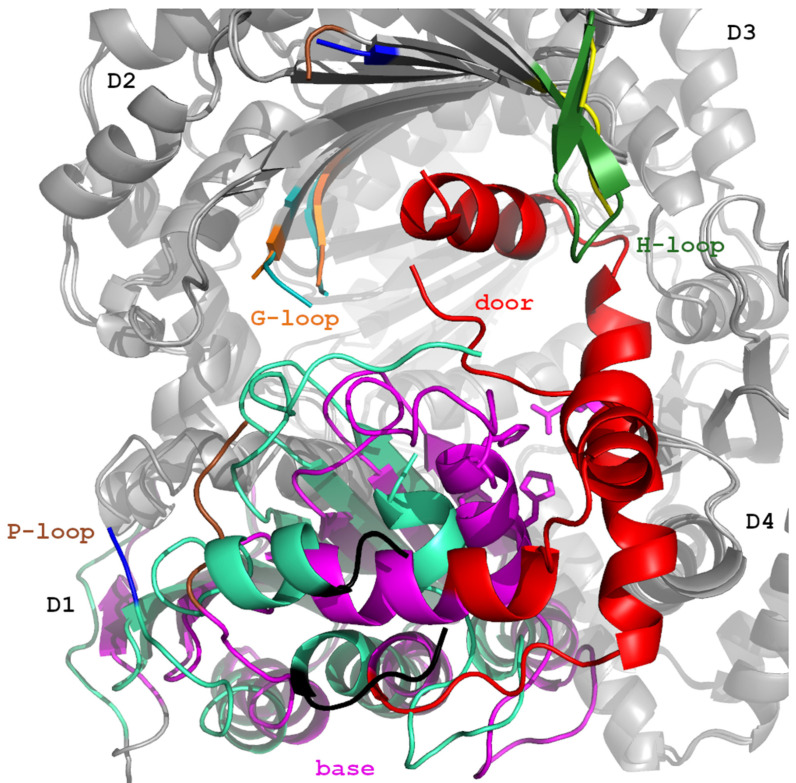
Comparison of IDE in the closed door and swinging door conformations. The base (including active site residues), door, P-loop, G-loop, and H-loop of IDE in closed door conformation are colored magenta, red, brown, orange, and dark green. Swinging door conformations are green-cyan, black, blue, cyan and yellow colors, respectively. Location of four subdomains (D1, D2, D3 and D4) is indicated. Figure was created using PyMol 1.3 and GIMP 3.0.6.

More details on the IDE catalytic mechanism were revealed by McCord et al., 2013 [45]. Comparison of different available crystal structures and SAXS analysis of IDE’s conformational states in solution revealed a relative rotation between IDE-N and IDE-C, the pivoting movement which allows IDE to open. This motion creates either D2/D3 or D1/D4 pivot states with opening on the opposite site of the molecule (increase in distance between domains D1/D4 and D2/D4, respectively) which allows the entry and degradation of larger substrates (e.g., amyloid β or insulin). Moreover, using an antibody fragment as a crystallization chaperone (Fab_H11-E_), it was possible to identify the third state, i.e., the swinging door state, which in combination with pivoting movement increases the number of IDE conformational states. The swinging door state is characterized by an 18 Å opening of the IDE catalytic chamber, permitting the entry of short peptides, like bradykinin or synthetic peptide substrate V. The key structural elements defining this state are located within the N-half of the enzyme, and they comprise the door and base subdomains and three loops (P-, H- and G-loop). The base subdomain contains the active site ^108^HFCEH^112^ motif, while the door subdomain includes the E189 residue, crucial for binding catalytic zinc, and a part of the hydrophobic exosite (Figure 6), which is important for the catalytic cleft formation. The door subdomain, P-, H-, and G-loop are not visible in the crystal structure, implying their multiple conformational states accompanied by the disruption of the catalytic site. The swing motion of IDE’s door subdomain is responsible for the opening of IDE’s catalytic chamber and together with the movement of highly mobile loops, controls IDE’s activity. An additional element of the mechanism is the body movement of the base subdomain toward G-loop and away from H-loop and D4 (Figure 9). The substrate binding blocks the swing motion, causing IDE chamber closure, which stabilizes the IDE catalytic cleft and allows substrate hydrolysis [45]. The stabilizing effect of the substrate binding on IDE’s structure was observed using hydrogen–deuterium exchange (HDX). Specifically, insulin stabilizes the entire IDE door subdomain, N-terminal exosite, and residues 821–830 in domain D4 that binds the P1’ and P2’ residues of IDE substrates after the scissile bond and H-loop [47]. The tight binding of IDE by Fab_H11-E_ stabilized the IDE door subdomain similarly to substrate binding and increased the catalytic activity of IDE by 50% [47]. Together, these studies reveal that IDE exists as a mixture of closed and open conformations in solution and has at least two types of motions: the swinging door in IDE D1 and pivoting between IDE-N and IDE-C.

### 5.2. Allosteric Regulation of IDE

The activity of IDE is regulated allosterically, and this enzyme exhibits a number of typical features of an allosteric protein, i.e., peptides increase the rate of hydrolysis of peptide substrates [114], and it shows kinetics characteristic for allosteric enzymes (i.e., sigmoidal shape of the kinetic curve and Hill coefficient equals ~2) [33,34,115,116,117,118,119,120]. The allosteric-like regulatory sites (distinct from the active site) were also indicated. Several sites in the IDE molecule are involved in allosteric regulation. (1) One of them is a place located within or close to the N-terminal exosite (the distal part of the substrate binding site) of the active subunit (Figure 6). The mutations V360S, I374S [33], and Y609F [33,120] blocked allosteric activation, as demonstrated by the altered enzyme kinetic profiles. (2) Certain residues of the ATP-binding site were also shown to be important in allosteric activation of IDE. The amino acid substitutions resulted in a decreased activation by the polyphosphate anions (K898A, K899A, S901A, and R429S, α12-α13 loop of D4 and D2, respectively) or deficiencies in activation by small peptides (K898A, K899A, S901A) [34]. (3) The intersubunit contacts play an important role in the allosteric regulation of IDE, as evidenced by a series of studies using various IDE variants of (a) different capabilities to form oligomers [119], (b) mixed IDE dimers containing combinations of mutations in active site (H112Q, H111F) or distal site (Y609F) in one or both subunits [120]. It has been proposed that appropriate subunit interactions are required for bound peptides to induce a conformational change in the same monomer and shift the equilibrium toward the open form [119]. (4) An additional element of IDE’s allosteric mechanism may be the hinge loop between IDE-N and IDE-C. A peptide bound to the N-terminal exosite lies close to the hinge region, and it also interacts with Y609 [33]. Similar proximity may be observed for the ATP-binding site of IDE (Figure 6). What is more, mutation F530A (part of the hinge) makes IDE hyperactive and alters its allosteric regulation [45]. It is believed that IDE’s allostery is mediated by the motion of the polypeptide chain in IDE, but the detailed mechanism is not known [47].

### 5.3. Oligomerization

Ability to form higher oligomeric assemblies may be another regulatory strategy of an enzyme. IDE forms various oligomeric structures in solution, dimers [22] or tetramers [114,121], but monomers were also observed [114,119]. The increase in concentration of IDE in solution as well as the presence of the substrate (bradykinin), shifted dimer-tetramer equilibrium towards the dimer; this was associated with a significant increase in the catalytic activity of the enzyme [114]. Monomerization mutations rendered IDE less active [119]; however, inorganic triphosphate (PPP_i_) and ATP were reported to shift the oligomeric state of IDE to monomers with a simultaneous increase in the rate of substrate hydrolysis [115]. Furthermore, analysis of cryo-EM IDE structures revealed preferred IDE O/pO state combinations, which indicate that IDE-N motions in the two subunits of the dimer are interdependent and allow substrate-induced closure of one subunit, leading to the transition of the other subunit to the open state. This enables the release of cleavage products or substrate uptake [47]. Oligomerization was also demonstrated to be required for IDE's allosteric regulation [119].

Some other M16 proteases also form oligomers, e.g., *Sphingomonas* sp. SPH2681/SPH2682 tetramer, consisting of two molecules each of SPH2681 and SPH2682 [26] and hPreP in the presence of the substrate [23], but many of them remain just monomeric, e.g., human PreP [23], *Escherichia coli* pitrilysin [122], *Thermus thermophiles* TTHA1264/TTHA1265 [28], and *Pectobacterium atrosepticum* FusC [29]. Hence, this regulatory strategy is not a rule among the M16 family members.

### 5.4. Modulation of IDE Activity and Substrate Specificity by Small Molecules

Features of the M16 protease catalytic chambers allow for cleavage of many different substrates, sometimes of opposing biological activity. These properties pose a problem in the development of therapeutic molecules designed to modulate the activity of M16 proteases, such as IDE. IDE’s main substrate, insulin, is a peptide hormone regulating glucose homeostasis, and the loss of proper insulin signaling causes diabetes. Inhibition of insulin degradation was long considered as a possible treatment strategy for type 2 diabetes [123,124]. This turned out to be difficult to implement as IDE degrades both insulin and glucagon, molecules decreasing and increasing glucose blood level, respectively [112]. Moreover, it also cuts amylin, amyloidogenic peptide, involved in the control of glucose homeostasis [37]. Several molecules were identified as affecting IDE’s activity in a substrate-specific way [41,42,44,125,126,127,128]. ATP, inorganic triphosphate [32,34,115], and high sodium chloride concentrations [34] were shown to accelerate the degradation of short peptides (e.g., a fluorogenic peptide derived from bradykinin-substrate V) by IDE. On the other hand, IDE’s activity against insulin was also shown to be inhibited by ATP [129] or no enhancement was found [115]. Nestin, an intermediate filament protein, was observed to inhibit the activity of IDE against ubiquitin [36], and both vimentin and nestin were shown to suppress IDE insulin-degrading activity. On the contrary, nestin enhanced the degradation of substrate V by IDE [125]. Binding of the crystallization chaperone (Fab_H11-E_) to IDE also enhanced the catalytic activity of IDE against substrate V [47]. As expected, in vitro, IDE is inhibited by metal ion-chelating agents, such as EDTA, 1,10-phenanthroline, and also by alkylating and thiol-modifying agents (e.g., p-chloro-mercuriphenyl-sulphonic acid (PCMPS), N-ethylmaleimide (NEM), iodoacetamide) [130]. It is also sensitive to hydrogen peroxide [35]. IDE is not inhibited by broad-spectrum hydroxamic acid inhibitors of conventional zinc metalloproteases [40]. Several specific inhibitors of high-potency binding to the active site were developed recently. Inhibitors Ii1 [40] and compound 1 bind to the catalytic zinc and lock it in a closed conformation [43].

**Figure 10:**
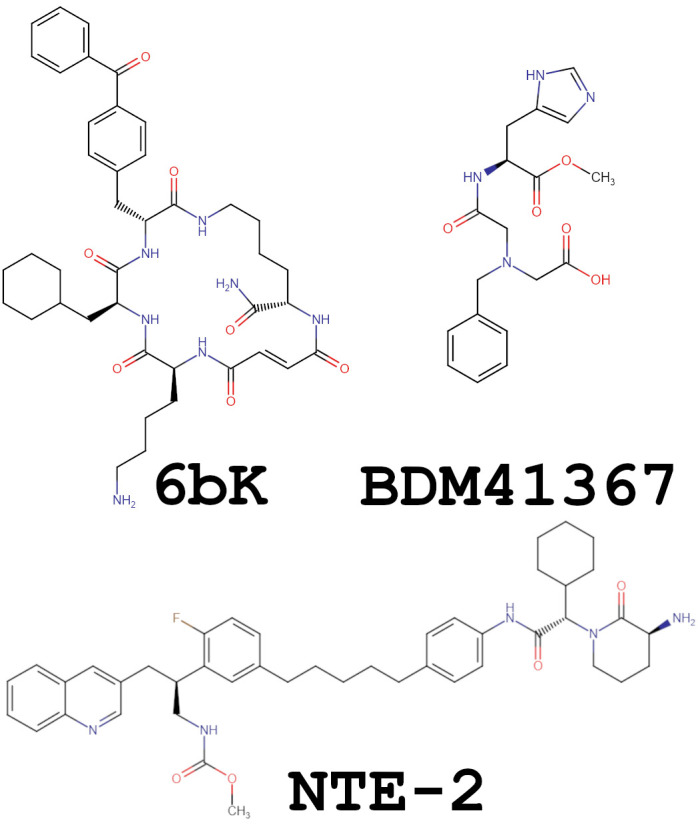
Structures of the representative IDE inhibitors. Two-dimensional structures of compounds 6bK (CID 91758793), BDM41367 (CID 53378106), and Nte-2 (CID 91801117) were obtained from Pubchem [131]. Figure was created using GIMP 3.0.6.

A promising method to selectively block IDE activity against specific substrates appears to be the use of compounds specific to exosites, either N-terminal exosite [33,103], hydrophobic exosite [103,112], or both exosites [103]. A good example is the 6bK inhibitor (Figure 10), which binds at the junction of domains 1 and 2 of IDE (the hydrophobic exosite), preventing substrate binding [112]. First, small ligands that bind to both the exosite and the catalytic site and modulate the proteolytic profile of IDE by altering its selectivity for amyloid β hydrolysis without affecting insulin in cellular models were BDM41367 (Figure 10) and its derivatives [41]. A compound with the opposite effect (BDM43079), which inhibits hydrolysis of amyloid β and promotes the hydrolysis of insulin, was also obtained [42]. A series of inhibitors designed by Maianti et al., 2019 demonstrated the ability to block insulin binding while allowing glucagon cleavage, opening up new possibilities for the development of IDE-targeted therapeutics [44]. Some of the inhibitors mentioned above were tested on various mouse models. Mice treated with 6bK experienced lower hypoglycemia and higher insulin levels compared to vehicle controls. Transient IDE inhibition improved blood glucose tolerance during oral glucose tolerance tests. Unfortunately, the results of the injected glucose tolerance tests were not as promising. An impaired glucose tolerance was observed, which was due to substantially higher levels not only of insulin but also of glucagon and amylin. Similar results were obtained for the compound 1 inhibitor [43]. Unfortunately, the results of in vivo studies were not always promising. For example, in a study in which dual-exosome binding inhibitors (NTE-1 and NTE-2, Figure 10) were developed, the results contrasted with those obtained using 6bK or compound 1, and a limited role for IDE in insulin clearance and action was found [103]. Therefore, further research is needed on the role of IDE in the regulation of blood glucose-regulating hormones and the development of more selective inhibitors.

## 6. Structure and Function of the M16B Exemplary Proteins: The Mitochondrial Processing Peptidases and the Core Proteins of the bc_1_ Complex

The mitochondrial processing peptidases (MPPs) and the core I and II proteins of the *bc*_1_ complex are evolutionary-related proteins. The MPP subunits are related to core proteins, where the α-subunit of the MPP is more similar to the core II protein, while the β-subunit resembles the core I protein [132]. However, the core proteins are usually not proteolytically active [48,62]. Interestingly, in plants, proteolytically active MPPs are part of the *bc*_1_ mitochondrial respiratory complex (discussed below). In yeasts and vertebrates, the MPPs are separate from the core proteins and are soluble and active in the mitochondrial matrix [132].

The MPPs are composed of two subunits, α and β, which are encoded by separate genes and are not connected by a linker [133]. β-MPP and α-MPP are referred to as Mas1 and Mas2 in yeast, and PMPCB and PMPCA in humans, respectively [134]. The heterodimer with a mass of ~100 kDa is composed of two ~500 residue subunits: (1) the catalytically active β-subunit with a zinc-binding HXXEH motif and (2) the homologous, non-peptidase α-subunit that lacks the zinc-binding motif, but it is still essential for the MPP function as it forms half of the enzyme cavity and contains a glycine-rich loop [7,135]. Yeast MPP is the only mitochondrial processing peptidase with a known crystal structure [20] (Figure 2B). The whole MPP cavity is strongly negatively charged, allowing for the formation of stabilizing electrostatic interactions with positively charged signal peptide substrates. Additionally, the highly polar cavity disfavors the amphiphilic α-helical structure of the substrate. Therefore, formation of β-sheets with the β4 strand of the MPP further extends substrate conformation aiding in proteolysis [20].

The *bc*_1_ complex (complex III or ubiquinol cytochrome *c* oxidoreductase or cytochrome *bc*_1_) is a structural and functional homodimer with a mass of ~460 kDa, consisting of a total of 10 to 11 subunits per monomer [68]. These include three subunits directly related to the electron transfer (cytochrome *b*, cytochrome *c*_1_, and the Rieske iron–sulfur protein) and seven subunits (QCR6, QCR7, QCR8, QCR9, QCR10, the core I and core II proteins) with no definite roles in the complex [136]. The core proteins are the largest accessory subunits of the *bc*_1_ complex; they account for more than one-third of the total mass of the complex and are exposed to the mitochondrial matrix [10].

There are multiple crystal or electron cryo-microscopy structures of the core proteins available within *bc*_1_ (Table 1). The crystal structures of core proteins as a part of the *bc*_1_ complex enabled the observation of their interaction with other subunits of *bc*_1_ in various organisms. The core proteins interact with the hydrophobic core of the *bc*_1_ complex, stably embedded in the inner mitochondrial membrane, but they are largely exposed to the mitochondrial matrix. Two *bc*_1_ complexes form a dimer in which the core subunits II and cytochrome b provide the main monomer–monomer contact surface in the matrix regions of the molecule. In mammals, subunit 6 (QCR6) also participates in the interaction between cytochrome b and both core proteins. Subunit 7 (QCR7) acts as a membrane anchor for the core protein I [11]. Another protein, subunit 9 (QCR9), is bound within the cavity formed by two core proteins mainly by hydrophobic interactions with β sheet of core II subunit [48]. In yeasts, the core II protein interacts with subunit 10 (QCR10) [69]. In plants, the structure of the *bc*_1_ complex exhibits a specific density corresponding to the zinc ion at the catalytic site of the β subunit of the component with the MPP activity. The extended N-termini of both MPP subunits extend across the dimer of the complex and provide additional contact with its membrane subunits [74].

The core proteins are involved in the formation of respiratory supercomplexes. The interaction of subunit 9 with the core II protein causes asymmetry of the *bc*_1_ complex, which in turn permits it to bind only one of the other respiratory complexes, namely complex I (CI) or IV (CIV), forming (*bc*_1_)_2_CI or (*bc*_1_)_2_CIV complexes, respectively. The factor responsible for the formation of (*bc*_1_)_2_CIV complexes is the SCAF1 protein. Its C-terminus is integrated with CIV, while the N-terminus is located inside one of the core protein cavities of (bc1)2 [81]. The interaction between CIV and (*bc*_1_)_2_ is strengthened further by the contact of COX7A and core I protein. In the case of (*bc*_1_)_2_CI complexes, contact between core I and subunit B22 (NDUFB9) of complex I is crucial for stabilizing the CI and CIII interaction [56,57]. Analysis of the ovine (*bc*_1_)_2_CI supercomplex revealed that subunit B15 (NDUFB4) also contributes to this interaction [79]. In yeasts, interactions between the core I and Cox5a have also been identified as necessary for (*bc*_1_)_2_CIV supercomplex [71]. In the *Arabidopsis* supercomplex, complexes I and (*bc*_1_)_2_ interact at three distinct sites of which one involves subunit B22 on the complex I side binding to the MPP-β and MPP-α of (*bc*_1_)_2_ complex [84].

In plants, *bc*_1_ complexes show an additional mitochondrial processing peptidase activity for the cleavage of mitochondrial-targeting signal peptides. Implications of this dual function of plant *bc*_1_ remain unknown [74], but the processing activity of the MPP is independent of electron transfer [134]. In other eukaryotes, the MPP is a separate soluble enzyme carrying enzymatic activity, e.g., in *Neurospora*, there is an addition of one homolog of MPP (α-MPP was probably duplicated from core II protein; bifunctional core I/β-MPP protein is required to exhibit proteolytic activity), and in yeasts and mammals, there are two [132]. Accessory subunits with the MPP homology have either lost MPP enzymatic activity (e.g., yeast) or retained basal activity (e.g., bovine homolog after additional detergent treatment of the complex) [137]. The Rieske iron–sulfur protein is the only known physiological substrate of mammalian core proteins [48,138].

## 7. Structure and Function of the Exemplary M16C Subfamily Members, Presequence Proteases

Presequence proteases belong to the M16C subfamily and are composed of a single polypeptide chain. These are mitochondrial and chloroplast proteins whose primary function is to degrade presequence peptides cleaved off from nuclear-encoded proteins. In addition, they also digest other aggregation-prone peptides [89]. Well-studied examples of these enzymes are human PreP (hPrep, metalloprotease 1, eupitrilysin) and *Arabidopsis thaliana* presequence peptidase (aPreP) [21], which are currently the only PreP proteins with determined crystal structures [23,89]. aPreP degrades peptides composed of 10 to 65 amino acid residues. These, besides presequence peptides, include a wide variety of other substrates: F1β subunit of the mitochondrial ATP synthase, the transit peptide of the small subunit of Rubisco, insulin B chain, galanin, penetratin, human prion protein peptide hPrPss, cecropin A, a synthetic peptide C1, and substrate V (bradykinin-derived) [108]. Human PreP was also shown to degrade non-presequence substrates, such as amyloid-β [111], dynorphin B-29, and other peptide substrates [109].

Based on the analysis of the crystal and cryo-EM structures of both peptidases, a mechanism of proteolysis by PreP was proposed. The aPreP structure in the closed state underlines the role of the extended hinge between two halves of the enzyme, which is characteristic of the M16C subfamily. In particular, hinge-bending motions are expected to mediate transition of the enzyme open state to the closed one in response to substrate binding. These motions bring two halves of the enzyme together, causing the two critical C-terminal residues (i.e., the R/Y pair) to approach the active site, completing it. Mutation of either residue results in the loss of proteolytic activity. The Tyr residue is believed to stabilize the transition state, lowering the energy needed for peptide bond hydrolysis, thereby increasing the reaction’s rate as seen for thermolysin [21]. More details are known about human PreP. Structural studies highlight the important role of the linker and point to the unique topography of the catalytic chamber, which allows for the specific capture and cleavage of diverse peptides with different distributions of charged and hydrophobic residues (discussed in Section 4.3). The cryo-EM studies [89] revealed three conformational states in substrate-free PreP, i.e., open, partially open, and partially closed states. The ligand-bound hPreP (PreP bound to amyloid β 1–40 or presequence peptide derived from human citrate synthase) existed in a distinct conformation, i.e., partially closed state. Comparison of these structures allowed to identify regions (designed as switches A, B, and C) which undergo substantial conformational changes during substrate hydrolysis. Regions A (D1-α4-α5, including extended loop with two β strands) and C (two α helices within halves linker region) move together with rigid body displacement between PreP-N and PreP-C and are characterized by high conformational dynamics. Furthermore, because switch A contains residues involved in substrate peptide binding and catalytic zinc ion coordination, it renders the partially open state incapable of catalysis. To stabilize the catalytic cleft and enable catalysis, substrate binding is required. Switch B represents α12 rotation axis and through residues H430 and E433 maintains contact with D3 of PreP-C (with residues N676-β5-α3, main chains of residues 642–643 of α2-β4 loop and R675-β5-α3, respectively). It also contributes residues to hPreP hydrophobic pocket (Figure 7) [89].

## 8. Structure and Function of the Protist M16 Metallopeptidases

Falcylisin (FLN) from *Plasmodium falciparum* is the best-studied example of the protist M16 family member. It belongs to the M16C subfamily of proteases and was identified as a promising target in antimalarial therapy [12]. The protein is found in digestive vacuole, apicoplast and the mitochondrion [12,13]. FLN is able to degrade apicoplast transit peptide and probably functions as an apicoplast presequence protease [139]. Additionally, it cleaves small oligopeptides which are products of upstream proteolytic degradation of hemoglobin, indicating the role of this protease in iron acquisition [12,13].

**Figure 11:**
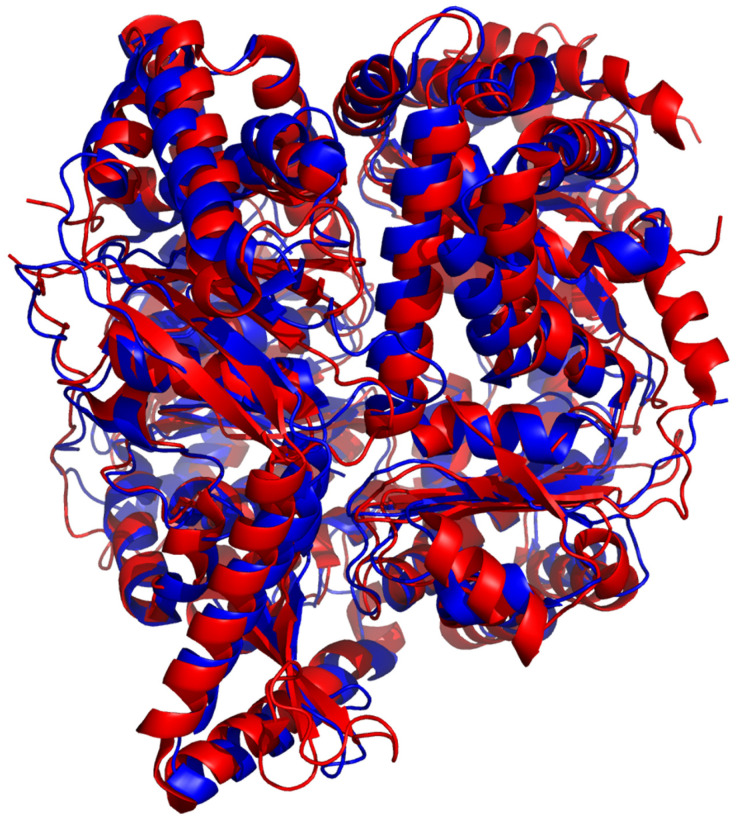
Superposition of human PreP and FLN. Structures of hPreP (6XOV, blue) and FLN (7DIJ, red) were aligned using PyMol with RMS over 4057 atoms: 1.932 Å. Figure was created using PyMol 1.3.

The crystal structure of FLN was solved (PDBid: 3S5I) and structures in complex with substrates [13] or inhibitors [12] are available. The structure of FLN superimposes well with eucaryotic M16C family members, e.g., with human PreP (Figure 11). FLN was identified as a common target of a subset of quinolines and other compounds working as antimalarial drugs. Five of these compounds inhibit FLN activity and were co-crystallized with FLN. The inhibitors were localized to a hydrophobic pocket of FLN (Figure 7), and this binding was speculated to lock the conformation of FLN in the closed form or to hamper the transition between the closed and open forms of FLN [12]. More details were obtained from FLN crystallized with peptide substrates derived from the hemoglobin α and β chains. The analysis of the crystals revealed two states, one representing a catalytically active closed state (β-peptide complex where the water molecule coordinating the zinc ion is absent) and the other, an inactive closed state (α-peptide complex where the zinc ion is tetrahedrally coordinated by H129, H133, E243, and a water molecule). Partially closed and open configurations were also observed in cryo-EM structures of free FLN and FLN preincubated with MK-4815 inhibitor, and the shift to the partially closed state was observed in the presence of MK-4815 [13].

Other examples of protist M16 proteases are INS-15, a metalloprotease potentially involved in the invasion of *Cryptosporidium parvum* [14,15,16], *Toxoplasma gondii* TLN4 that cleaves β-insulin and plays an important function in the process of *Toxoplasma gondii* egress from host cells [17], or *Plasmodium falciparum*, a stromal-processing peptidase (SPP) homolog which cleaves the transit peptide of an apicoplast-targeted protein upon the import of the protein into the apicoplast [140].

## 9. Prokaryotic M16 Proteins

Representatives of the M16 family have also been identified in prokaryotes, but data on their specific cellular functions are scarce, and their physiological substrates are poorly defined. Examples of M16 procaryotic family members, their function, and substrates are shown in Table 2.

**Figure 12:**
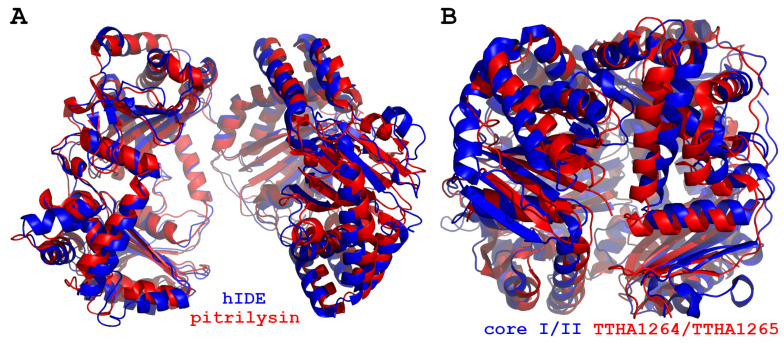
Superposition of M16A and M16B family representatives. (**A**) Human IDE (6BF8, blue, open apo-state) was aligned with pitrilysin (1Q2L, red) using PyMol with RMS over 5156 atoms equal to 2.512 Å. (**B**) Bovine core proteins I/II (2A06, blue) were aligned with TTHA1264/TTHA1265 (7V4Y) proteins (red) with RMS over 3441 atoms equal to 3.492 Å. The figure was created using PyMol 1.3 and GIMP 3.0.6.

Crystal structures of a few procaryotic M16 proteases were solved, and they show relatively high structural similarity to eucaryotic members of this family (Figure 12). *Bacillus halodurans* BHP is the M16B protease, and it shares the clam shell architecture common to the M16 family peptidases, except it forms stable homodimers (while a majority of M16B proteases are heterodimers). Both open (without substrate) and closed (with substrate) conformations of the enzyme were revealed using SAXS technique. Modeling of the open-state dimer showed that it resembles its eukaryotic M16B orthologs, and cycling between open and closed states was suggested to rely on the rotation of an axis running close to the long axes of the helix α12. This movement requires the maintenance of an extensive interaction interface between the BHP monomers. Substrate binding induces complete domain closure which is required for catalytic activity [25]. It is worth mentioning that the rotation of α12 was also observed for human PreP [89].

Other examples of prokaryotic M16 proteases are pairs TTHA1264/TTHA1265, SPH2681/SPH2682 and AlbF/AlbE. TTHA1264 was identified in *Thermus thermophiles* HB8, and it was crystallized as an open homodimer; however, in solution, it was monomeric and inactive [88]. It was later predicted that the neighboring gene, TTHA1265, encodes its partner, with which it forms the active heterodimer [25]. The SEC-MALS analysis confirmed the formation of a stable heterodimer in solution, while crystallography provided the structure of the TTHA1264/TTHA1265 heterodimer complex [28]. TTHA1264/TTHA1265 shares high structural similarity with BHP and *Sphingomonas* sp., A1 protease SPH2681/SPH2682, but has a significantly more open conformation. A larger opening of TTHA1264/TTHA1265 is possible because a smaller contact area and fewer interactions between residues at the monomer interface have been demonstrated [28]. SPH2681/SPH2682 also form active heterodimers, and crystallography revealed both open and closed conformations within the same crystal. Comparison of these conformations allowed to observe the rigid body rotation of SPH2681/SPH2682 around the edge of the molecule located on the opposite side of the zinc ion-binding motif. SPH2681/SPH2682 in the closed form largely overlaps BHP homodimer; however, when compared to MPP, the SPH2681/SPH2682 opening was substantially smaller as it does not contain glycine-rich loop [26]. Another example of prokaryotic M16 proteases with known crystal structure is AlbF/AlbE, and it has an open conformation with an opening wider than MPP. AlbF/AlbE has a wider and shallower substrate-binding chamber in comparison to other M16 proteases due to shorter β strands in D2 of AlbF. These characteristics contribute to the increased affinity of AlbF/AlbE for the subtilosin precursor and maintenance of precursor conformation during reactions. Furthermore, a hypothesis of the existence of a channel connecting the active site with the protein surface has been put forward, as analysis of the structure of SPH2681/SPH2682 in the closed form resulted in the detection of a similar channel. These features allowed to propose a model in which the subtilosin precursor is first bound, then the leader peptide is cleaved and released through the channel, and, finally, the mature subtilosin is released after its macrocyclization. Nonetheless, the detailed chemical mechanism for macrocyclization, as it is not a typical activity of M16 proteases, and the structure of AlbF/AlbE with the substrate peptide are still lacking [27].

Other, atypical members of the M16B subfamily with solved crystal structure are also known, i.e., *Pectobacterium atrosepticum* FusC and its *Escherichia coli* homolog PqqL [29,30]. Both proteins are examples of M16B, but their active molecules are single polypeptides; hence, the halves are connected by hinges as in M16A or M16C subfamilies. Analysis of the crystal structure of FusC in complex with its substrate, ferredoxin, allowed to identify two sites. The first of them, the F1, binds the C-terminal fragment of the substrate. It contains the residues Y298 (α9), M392 (α12), and A428 (α14) that build a hydrophobic pocket corresponding to the human PreP or FLN hydrophobic site (Figure 7). The second site, referred to as F2, close to sites corresponding to IDE exosites, binds the N-terminal residues of ferredoxin. The middle part of ferredoxin was not visible most probably due to its partial unfolding. The bound ferredoxin was intact [29]; however, it has been shown that FusC can degrade ferredoxin [145], confirming the role of this peptidase in the transport of ferredoxin across the membrane and its further processing. The structure of PqqL is analogous to FusC. PqqL was observed in open conformation in the crystals, contrary to closed conformation of FusC. Using molecular dynamics simulations and SAXS, additional conformational states, not visible in crystal structures, were observed for both proteins. These include a range of intermediate conformations, among others, closed and partially closed for PqqL and fully open state for FusC [30].

## 10. Conclusions

M16 proteases are a family of metallopeptidases with a characteristic bi-modular clam shell-like structure. The presence of both modules (halves of the molecule) is necessary to achieve full activity, proper catalysis regulation, and substrate specificity. This is due to the distribution of residues forming the active center and other catalytically important sites, to which both halves must contribute. It is important to highlight substrate binding sites (exosites), ATP-binding site, area of intersubunit contact, and the hinge connecting N- and C-halves of the enzyme, as all of them can be important for allosteric regulations of those proteases. The connections between the halves, linkers which serve as hinges, allowing the adoption of an open or closed conformation, are also crucial for activity.

Despite the very similar structure of the active molecule, two functional classes can be distinguished in this family: peptidasomes and processing peptidases. They differ not only in the type of substrates they recognize but also in their mechanism of action. Peptidases enclose peptide substrates of a specific size in a central chamber and degrade them while their structure undergoes cycles of open and closed conformations. On the other hand, processing peptidases primarily cleave N-terminal peptides at more strictly defined sites, while an additional structural element, glycine-rich loop, keeps the enzyme conformation partially open.

The diversity of substrates recognized and processed by individual M16 peptidases significantly hampers the specific modulation of their activity. However, a more detailed understanding of their regulatory mechanisms (especially those based on allostery) and the sites responsible for the specific binding of individual substrates in the catalytic chamber may in the future enable the design of modulatory compounds specific to selected substrates. This may lead to the development of new strategies to counteract the development of Alzheimer’s disease and diabetes or combat infection-related diseases.

## Figures and Tables

**Table 1:** Summary of members of M16 family proteases with available structures.

#	Name	MEROPS	Uniprot (Organism)	aa	kDa	Active Site HXXEH and Distal Glu	Available Structures
1	Pitrilysin	M16A	P05458 (*Escherichia coli*)	962	107.7	^88^HYLEH^92^-E^169^	PDB id: 1Q2L
2	PqqF	M16A	A0A1A9TAI5 (*Serratia* sp. (strain FS14))	773	84.5	^51^HLLEH^55^-E^132^	substrate-free [31]
3	human Insulysin (hIDE)	M16A	P14735 (*Homo sapiens*)	1019	117.9	^108^HFCEH112-E^189^	substrate-free [32], complex with substrates (insulin B chain, amyloid-β peptide (1–40), amylin and glucagon) [22], rat IDE [33,34], IDE complexed with other protein or peptide substrates: bradykinin [35], ubiquitin [36], IGF-II and TGF-α [37], MIP-1/CCL3 [38] or CFE111Q/CCL4 [39], with activators, like ATP [33], or inhibitors [40,41,42,43,44], fab-bound [45,46,47], IDE open structure [47].
4	rat Insulysin (rIDE)	M16A	P35559 (*Rattus norvegicus*)	1019	117.7	^108^HFCEH^112^-E^189^	
5	mitochondrial processing peptidase (MPP)	M16B	P10507/P11914 (*Saccharomyces cerevisiae*)	462/482	51/53.3	^70^HFLEH^74^-E^150^	substrate-free and in complex with substrates [20]
6	bovine Core I/Core II	M16B	P31800/P23004 (*Bos taurus*)	480/453	52.7/48.1	^91^YFVEH^95^-E^171^	bovine [11,48,49,50,51,52,53,54,55,56,57,58,59,60,61], yeast [62,63,64,65,66,67,68,69,70,71,72,73], mung bean [74], chicken [75,76,77], ovine [78,79], human [80], mouse [81,82,83], *Arabidopsis thaliana* [84] or *Tetrahymena thermophila* [85,86,87].
7	yeast Core I/Core II	M16B	P07256/P07257 (*Saccharomyces cerevisiae*)	457/368	50.2/40.4	not detected	
8	mung bean Core I/Core II	M16B	A0A1S3TWG4/A0A1S3VF71(*Vigna radiata* var. radiata)	527/506	58.6/54.4	^137^HFLEH^141^-E^217^	
9	BH2405 (BHP)	M16B	Q9KA85 (*Halalkalibacterium halodurans*)	413	46.4	^46^HFLEH^50^-E^126^	complex with peptide ligand [25]
10	TTHA1264/TTHA1265	M16B	Q5SIV0/Q5SIU9 (*Thermus thermophilus*)	406/403	45.5/43.8	^46^HFLEH50-E^125^	substrate-free [28,88]
11	SPH2681/SPH2682	M16B	F2Z284/F2Z283 (*Sphingomonas* sp. strain A1)	437/424	48.3/45.8	^51^HALEH^55^-E^131^	substrate-free [26]
12	AlbF/AlbE	M16B	A0A9X9ZA61/A0A9X9ZA64 (*Bacillus thermotolerans*)	366/381	42.5/44	^54^HFLEH^58^-E^130^	substrate-free and with bound Ni^2+^ [27]
13	FusC	M16B	Q6D8U3 (*Pectobacterium atrosepticum* SCRI1043)	924	103.9	^80^HMVEH^84^-E^165^	complex with substrate (ferrodoxin) [29]
14	PqqL	M16B	P31828 (*Escherichia coli*)	931	104.6	^80^HFVEH^84^-E^167^	substrate-free [30]
15	falcylisin (FLN)	M16C	Q76NL8 (*Plasmodium falciparum*)	1193	138.8	^129^HILEH^133^-E^243^	complex with substrate (hemoglobin) [13] or inhibitors [12]
16	human presequence peptidase (hPreP)	M16C	Q5JRX3 (*Homo sapiens*)	1037	117.4	^104^HILEH^108^-E^205^	substrate-free and in complex with substrate or peptide ligand [23,89]
17	arabidopsis presequence peptidase (aPreP)	M16C	Q9LJL3 (*Arabidopsis thaliana*)	1080	121	^162^HILEH^166^-E^262^	complex with peptide ligand [21]

**Table 2:** Summary of exemplary procaryotic M16 family members.

Protease Name (Organism)	M16 Protease Subfamily	Description/Function	Tested Substrates
pitrilysin (*Escherichia coli)*	M16A	located in the periplasmic space, degrades β-galactosidase, function unknown [141]	insulin, secretin, vasoactive intestinal peptide, thyrocalcitronin, substance P, angiotensinogen, luteinizing hormone-releasing hormone peptide and synthetic substrate QF27 [142]
PqqF (*Serratia* sp.)	M16A	cleaving two residues, glutamate and tyrosine from PqqA which are subsequently used for coenzyme pyrroloquinoline quinone (PQQ) biosynthesis [31],	n/a
hrrP (*Sinorhizobium meliloti*)	M16A	encoded on accessory plasmid, capable of degrading a range of nodule-specific cysteine-rich peptides used by *Medicago truncatula* for inducing and maintaining rhizobial differentiation [143]	n/a
SidC *(Vibrio vulnificus)*	M16A/C	degrades insulin and glucagon affecting glucose levels in infected mice [144]	n/a
FusC (*Pectobacterium atrosepticum)*	M16B	periplasmic, used to acquire iron [29]	ferredoxin [145]
PqqL (*Escherichia coli)*	M16B	periplasmic, used to acquire iron [30]	n/a
BHP (BH2405, YmxG peptidase, *Bacillus halodurans)* [25]	M16B	n/a	n/a
TTHA1264/TTHA1265 (*Thermus thermophilus)* [28]	M16B	n/a	n/a
SPH2682/2681 (*Sphingomonas* sp., A1)	M16B	n/a	endorphin, insulin, dynorphin A (1–13), Leu-enkephalin and bradykinin [26]
AlbF/AlbE (*Quasibacillus thermotolerans)*	M16B	involved in maturation of subtilosin, a bacteriocin exhibiting antibacterial activity against Gram-negative and Gram-positive bacteria [27]	n/a
ppBH4 (*Bacillus halodurans)*	M16B	n/a	insulin, neurotensin, dynorphin A, kemptide, leucine-enkephalin [146]
RPP *(Rickettsia prowazekii)*	M16B	n/a	preferring positively charged peptides like dymorphin A; vasoactive intestinal peptide; mastoparan; and αMSH amide but also mitochondrial presequence peptides [147]
PqqE (HP1012, *Helicobacter pylori)*	M16B	junctional adhesion molecule A (JAM-A) disrupting gastric epithelial integrity [148]	n/a

n/a—not available.

## Data Availability

No new data were created or analyzed in this study.

## Abbreviations

The following abbreviations are used in this manuscript:

cryo-EM	cryo-electron microscopy
FLN	falcylisin
GRL	glycine-rich loop
HXXEH	amino acid sequence: His-X-X-Glu-His, X–any amino acid
IDE	insulin-degrading enzyme
MPP	mitochondrial processing peptidases
PreP	presequence peptidases
R/Y pair motif	Arg/Tyr pair motif
SAXS	small-angle X-ray scattering
